# Design and Calibration of Multi-Featured Large H-Coil Sensor Intended for 2D and Rotational Measurements

**DOI:** 10.3390/s26154726

**Published:** 2026-07-25

**Authors:** Stan Zurek, Dimitar Petsov

**Affiliations:** 1 Megger Instruments Ltd., Archcliffe Road, Dover CT17 9EN, UK; dp565@kent.ac.uk; 2 School of Engineering, Mathematics and Physics, University of Kent, Canterbury CT2 7NZ, UK

**Keywords:** H-coil, H sensor, B-coil, rotational magnetisation measurements, 2D measurements, rotational measurements, rotational power loss, tangential magnetic field strength

## Abstract

**Highlights:**

This paper describes the prototyping of a large H-coil for measuring the tangential magnetic field strength *H* intended for 2D or rotational measurements. Multiple features are demonstrated simultaneously by using PCB design. Sensitivity was calculated from the designed dimensions and agreed very well with verification (calibration) in a long solenoid.

**What are the main findings?**

- It is possible to accommodate the space for B-coil wires by using a sectioned H-coil design, whilst eliminating the sensitivity to the *H_Z_* component.
- Parasitic self-capacitance is estimated from experiments.

**What are the implications of the main findings?**

- It is postulated that better spatial averaging is going to be achieved with such a large coil.
- Measurement bandwidth is not affected up to 100 kHz despite the relatively large self-capacitance.

**Abstract:**

Dual H-coils are used for 2D and rotational measurements of magnetic field strength *H*. For precise measurements, they need to be constructed with a high level of orthogonality, which can be achieved by using a PCB (printed circuit board). This technology was employed for designing and manufacturing a multi-feature H-coil with the following functions: two sensing coils (X and Y) on the same large PCB substrate (165 mm diagonal), controlled orthogonality for each turn of the sensing coils, suppressed sensitivity to the *H*_Z_ component by eliminating the off-axis active area, thin former (0.75 mm nominal), sectioned design for accommodating B-coil wires so that the H-coil can be placed directly on the sample surface (demonstrated for the first time in the literature), stackable design enabling extrapolation of *H* towards sample surface, estimation of the order of magnitude of self-capacitance (demonstrated for the H-coils for the first time) and bandwidth (shown experimentally to hold up to 100 kHz), verification of sensitivity (calibration) in a long solenoid. Good agreement was obtained between sensitivity estimated from dimensions and from calibration (2% or better). The multiple features demonstrate the state-of-the-art collection of improvements as suggested separately in the literature before.

## 1. Introduction

H-coils belong to a class of inductive sensors [1,2] with the intended use of sensing magnetic field strength *H*, measured in (A/m). Despite its name, an H-coil actually senses variation in flux density *B* (T) penetrating it, as dictated by Faraday’s law of induction. This can be expressed by the changing magnetic flux *Φ* (Wb), or flux linkage λ (Wb), and is also linked to the penetrating *H*. Internal electromotive force *V_EMF_* (V) is induced in the sensing coil with *N* turns (unitless) and active cross-sectional area *A* (m^2^).

(1) $$V_{EMF}=-\frac{d\lambda }{dt}=-N\frac{d\Phi }{dt}=-NA\frac{dB}{dt}=-NA\mu \frac{dH}{dt}$$

Therefore, a linear relationship is required between *B* and *H* of the substrate material over which the sensing coil is disposed, so that the magnetic permeability *μ* (H/m) in Equation (1) is constant. For typical paramagnetic materials, the relative permeability *μ_r_* (unitless) is linear and negligibly close to unity (as compared to other factors), and thus the absolute permeability is close to that of vacuum’s *μ*_0_ (H/m):

(2) $$B=\mu_{r}\mu_{0}H\approx \mu_{0}H$$

So under such conditions, the linearity of *H* in time *t* (s) can be utilized as in Equation (3). For the full transducing equation, it is also necessary to perform an integral as per Equation (4), either in the analogue or digital domain [2,3].

(3) $$H(t)\approx \frac{B(t)}{\mu_{0}}$$

An important point to consider is that the internal electromotive force *V_EMF_* is not accessible. Instead, the quantity that is measured is the output voltage *V_H_* of the H-coil, but because it is a quantity that is measured by an external device, its sign is reversed [3], as represented by the *positive* contribution of the *measured V_H_*(t) in Equation (4). For symmetrical periodic signals, the constant can be subtracted away, as the signal must be oscillating around zero, due to Equation (1), which forbids any DC contribution.

(4) $$H\left(t\right)=\frac{1}{\mu_{0}NA}\int V_{H}\left(t\right)dt+const.$$

Equation (4) forms the basis for using an H-coil as a sensor of magnetic field strength *H*. However, in the general case of a manufactured coil, the *NA* product in the denominator is not known *precisely* (as also demonstrated experimentally below), and thus a suitable calibration procedure is typically applied so that the sensitivity can be defined accurately. Note that such a calibration constant can be expressed in a number of ways, depending on whether it includes the reciprocal factor, just the *NA* value, or also the *μ*_0_ component, or even the frequency of calibration, so the numerical values used in a given system can vary widely by many orders of magnitude. Their use should be clear from the context.

In a hypothetical case, increasing *μ* (Equation (1)) would directly increase sensitivity. But in practice, using ferromagnetic materials would harm sensing linearity, and thus only non-magnetic substrates are used. H-coils can be exposed to a wide range of amplitudes (e.g., up to 40 kA/m) [3], so magnetic saturation could occur if a ferromagnetic material was present.

**Figure 1:**
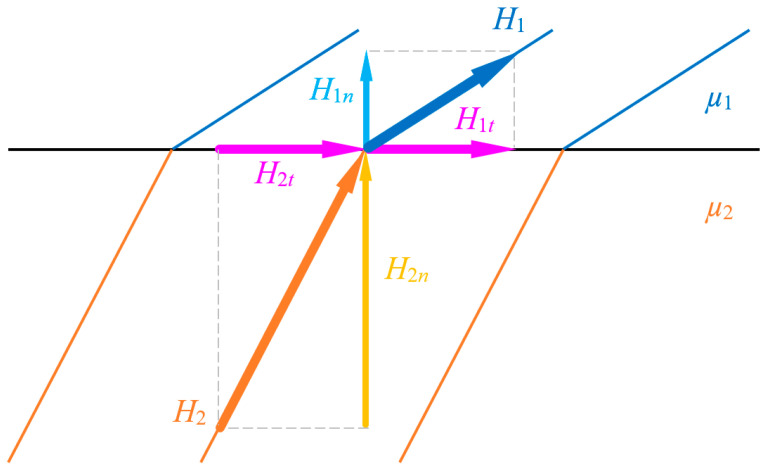
Equality of tangential *H* so that *H*_1*t*_ = *H*_2*t*_ at the boundary of two materials with permeabilities *μ*_1_ and *μ*_2_.

The additional assumption is the equality between the values of tangential *H_t_* at the boundary of two materials [2,3,4,5,6,7,8,9,10,11,12,13,14,15,16,17,18] (Figure 1). Thus, the value of tangential *H*_2*t*_ immediately under the surface of the sample under test can be detected with a suitable sensor placed just outside of that surface, so that *H*_1*t*_ is measured, and thus *H*_2*t*_ ≈ *H*_1*t*_ ≈ *H_t_*. For lower frequencies and thin samples of typical laminated magnetic materials (sheets or ribbons), *H* inside the material can be assumed to be uniform. This may not be true anymore at higher frequencies due to the skin effect, where also other processes will take place, but this topic is outside of the scope of this paper.

Also, in order to exploit Equation (4) for sensing, yet another assumption is made that the to-be-measured *H* penetrating the H-coil is spatially uniform. For a contiguous H-coil with uniform winding and thin design, it could be argued that the *H* uniformity is not strictly necessary, because such a coil would behave similarly to a Rogowski–Chattock potentiometer, by having its ends close to the sample surface [2,3,4]. But for an H-coil which does not have uniform or contiguous winding, this assumption no longer holds, and thus *H* uniformity is required.

In this project report, a multi-featured large dual H-coil with interleaved topology was designed, manufactured as a printed circuit board (PCB), and verified (calibrated) in a solenoid. The paper focuses on several distinct features implemented *simultaneously* in this design:

- Two sensing coils on the same PCB, with controlled orthogonality.
- Interleaved sensing coils (for *H_X_* and *H_Y_*) within the same PCB.
- Suppressed sensitivity to the normal component of *H* (namely *H_Z_*).
- Thin rigid former (nominal 0.75 mm).
- Sectioned design for accommodation of B-coil wires without sacrificing distance to the sample surface—demonstrated for the first time in the literature.
- Large format (up to 116 mm side, 165 mm diagonal) for improved spatial averaging of measured values.
- Stackable design for the possibility of extrapolation of *H* towards the sample surface.
- Estimated self-capacitance of the H-coil by itself, and as placed on the surface of a metallic sample (attempted for the H-coils for the first time in the literature).
- Verification of sensitivity (calibration) in a uniform magnetic field of a long solenoid.

It should be emphasized that this study focuses on the design of the dual H-coil itself, which is intended to be used in an improved 2D and rotational magnetization system. The topic of measurement of 2D or rotational properties with such H-coils is beyond the scope of this paper, but it will be tackled in future research within this project.

## 2. Dual Orthogonal Sensing Coils

**Figure 2:**
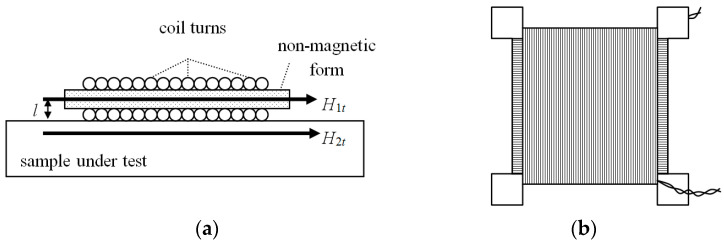
Example of an H-coil for measurement of tangential *H*: (**a**) H-coil placed on the surface of the sample under test. (**b**) Concept of a wire-wound dual H-coil with both coils disposed around the same former, so that two orthogonal components of *H* can be sensed simultaneously (*H_X_* and *H_Y_*).

The tangential *H* does not remain equal in amplitude at higher elevations from the sample surface, and thus a certain amount of gradient can be expected, as indeed proved experimentally by using stacked H-coils [3,4,5]. It is thus desirable for the H-coil to be as thin as possible so that the mismatch between the real *H* directly at the sample surface and the actual sensing axis of the H-coil (positioned at an effective distance *l*, Figure 2a) is minimized. Alas, some disparity will always be present, because it is not possible to fabricate a realistic H-coil with zero thickness—such a coil would have zero sensitivity, due to *A* = 0 (as per Equation (1)).

For uni-directional measurements, the angular positioning is less critical due to the usually negligibly small cosine error even for large angular misalignment. For example, even a relatively large misalignment of 4° away from the ideal axis will produce a magnitude error of only 0.25%, whereas 1° would produce a negligibly small error of only 0.015% (as compared to other sources of uncertainty). Thus, control of orthogonality is less important.

This is not the case for 2D measurements (rotational and alternating), for which even a small angular sensor misalignment can significantly affect the measured value of power loss, with experimental results proving that for some measurement systems and samples under test a misalignment smaller than 0.5° can result in *negative* readings of power loss [3,6,7]. Consequently, averaging from clockwise/anticlockwise direction (of rotation), or positive/negative inclination of *H* (in 2D measurements) must be applied, as otherwise large errors can occur [3,6,10,13].

Dual wire-wound dual H-coils (Figure 2b) can be wound on the same former, as practiced by multiple international researchers for rotational and 2D measurements over the decades [1,2,3,4,5,6,7,8,9,10,11,12,13,14,15,16,17,18]. The advantage of such a design is that the coils are disposed around the same former and thus their axes of sensitivity coincide within the same X-Y plane, i.e., at the same effective elevation *l* in the Z direction (Figure 2a), because the outer coil is wound around the inner coil. However, the inner coil will have a smaller active cross-sectional area and thus somewhat lower sensitivity. But more importantly, it is difficult to control *precisely* the wire positioning, which leads to uncontrolled and unknown non-orthogonal effective positioning of the X and Y sensing axes, especially for smaller coils. Even when robotic winding is used [9], the absolute wire positioning cannot be guaranteed, as it is dictated to some extent by the local surface roughness of the substrate. Multiple layers of winding compound the problem, and so do the small dimensions of the former, which will contribute additional angular positioning errors due to mechanical mounting tolerances.

**Figure 3:**
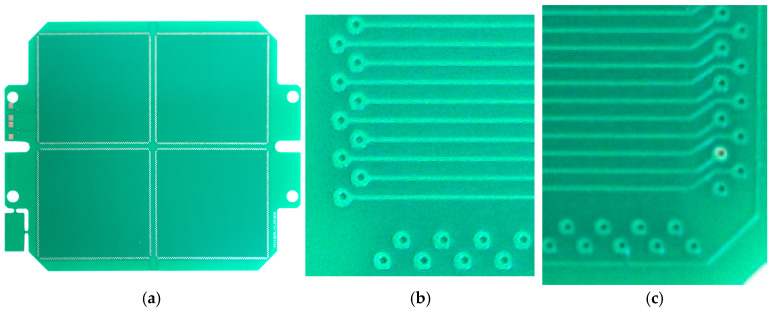
Photographs of the investigated prototype: (**a**) as-made whole dual H-coil with the sacrificial rectangle in the bottom left corner. (**b**) Close-up showing the parallel tracks and spacing between vias. (**c**) Details of track positioning at the other end of the coil with the return track also visible on the outside. The photographs are taken before further mechanical processing.

For PCBs, the positioning of each “wire” can be controlled to a much better degree. By using standard PCB technology (as employed in the investigated prototype), the basic sensitivity is reduced due to lower density of turns that can be accommodated over the same unit length, as compared to the wire-wound case. This is dictated by the increased size of the vias and the required minimum separation distance between the PCB tracks, as required by the etching process during fabrication. In this case, it was 0.15 mm for the track width and spacing, and 0.25 mm diameter for each circular via pad (Figure 3). However, sensitivity is compensated by larger dimensions, and also a high number of turns is not necessarily advantageous because increased inductance could lead to limitation of sensing bandwidth, as discussed below.

**Figure 4:**
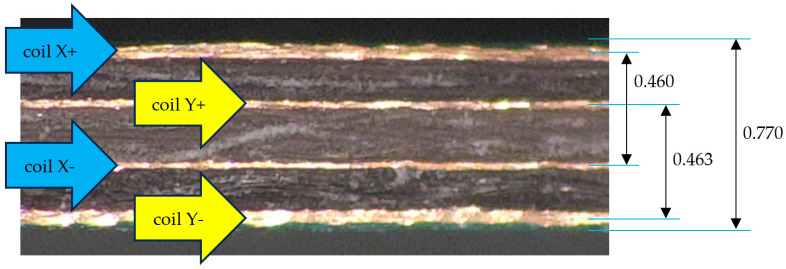
Cross-section through the PCB, with estimated dimensions. Plus and minus denote the “to” and “from” layers. The dimensions are given in mm.

The PCB has four copper layers, and the coils are interleaved within the same substrate so that the coil X occupies layers one and three (counting from the top in Figure 4), and the Y coil occupies layers two and four. Such interleaving is not possible with wire-wound coils, which would have to occupy layers one and four for the first coil, and two and three for the second coil. The drawback is that, for such a PCB coil, the sensing axes will not lie in the same plane and will consequently measure *H* at a different level in the gradient, thus possibly contributing to some level of systematic error. So alternatively, the coils could have been made as X in layers 1–4 and Y in layers 2–3, which would mimic a wire-wound coil and achieve “the same sensing plane” at which the respective *H*-vector component will be measured.

However, the design was executed in such a way that two PCB-based coils can be stacked on top of each other, with their dimensions known to a reasonable precision. Therefore, extrapolation of the measured *H* waveforms can be conducted on a point-by-point basis in the time domain of the *H* waveforms, as published in the literature of the subject [2,3,5,10], and thus the systematic errors due to such a gradient can be significantly reduced (practically eliminated). Hence, the difference in the sensing plane locations is not critical with such an approach. This is also discussed in more detail in Section 5.

The cross-section shown in Figure 4 was made on the small sacrificial rectangular PCB piece visible in the bottom left corner of Figure 3a. The nominal designated PCB thickness was 0.75 mm, but the actual total thickness measured for the manufactured PCB was 0.770 mm, mostly because of the additional coating that is applied on the surface (such as the solder mask layer). A cross section was executed by cutting out the sacrificial piece, cutting through it and polishing the cross section, and taking a photograph under a microscope. The total measured thickness was used as a reference value, and the sub-coil dimensions were estimated from the microphotograph (as shown in Figure 4), so that the centre of each conductor was used for the scaled value for calculation. This was performed at three places in the image and averaged separately for X and Y coils, as shown in Figure 4. This approach was justified because it is used here only to demonstrate the estimated values, as the independent calibration was used for the definition of the effective cross-sectional area.

The total sensing width of the coil (ignoring the cross-shaped cutout) is measured as an average between the line straddling the two rows of vias for each sub-coil (X and Y).

## 3. Suppressed Sensitivity of the Z Component

**Figure 5:**
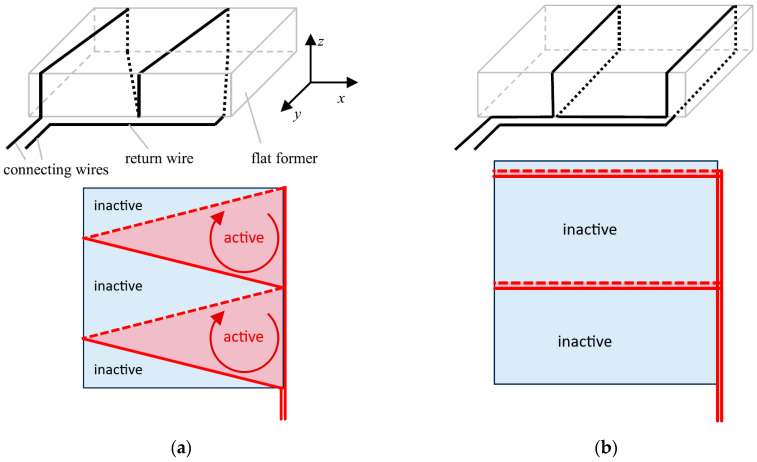
The concept of unwanted sensitivity in the Z axis (simplified diagram): (**a**) for a wire-wound H-coil; (**b**) for a PCB-based H-coil (with the active area eliminated or reduced to very narrow strips). The solid lines denote wire fragments visible from the line of sight, and the dashed lines those hidden by the flat former. Upper images show isometric view, bottom images show top view.

The sensitivity of H-coils to the Z component (perpendicular to the sample surface) is rarely discussed in the literature by other researchers, but the effect is certainly present and measurable, especially for magnetically open systems (with an air gap) [15,16]. Wire-wound H-coils have the wire disposed helically around the former, so when looking from the top there is a non-negligible unwanted active area of sensitivity to the *H_Z_* component (Figure 5a). The actual area also depends on how the return wire is positioned, but it does not depend on the number of turns [15]. This area can be eliminated or at least significantly reduced if the subsequent turns are kept exactly on top of each other, in a specific parallel/perpendicular fashion (Figure 5b) [16].

Notably, the return conductor is positioned so that it does not enclose additional area. This approach was applied for the sub-coils in the prototype described in this study, which is especially important for the multi-section design, as explained below.

It should be emphasized that, with the approach as conceptually illustrated in Figure 5b and executed with this prototype, the positioning and orthogonality of each and every turn is controlled, rather than relying on relatively random local positioning of wire in a helical winding. For wire-wound H-coils, the non-orthogonality can be at the level of 0.6–1.8° [9,17]. However, for the PCB-based coil, better overall sensing orthogonality should be possible to expect because the positioning of each turn does not rely on random chance but on precise design.

Regrettably, quantification of orthogonality was not attempted by the authors, as it is very difficult to measure it precisely [3,17]. For this study, the size of the available solenoid as the source of a uniform magnetic field (described in the next sections) was not large enough to accommodate rotation of the coil, which is necessary for such an orthogonality test. Additionally, to exclude any eddy current effects, ideally the turntable would have to be made out of non-magnetic and non-conductive materials, which are difficult to machine precisely. This exercise was beyond the topic of this project update, but it may be attempted in the future if suitable equipment becomes available.

## 4. Sectioned Design for Accommodation of B-Coil Wires

**Figure 6:**
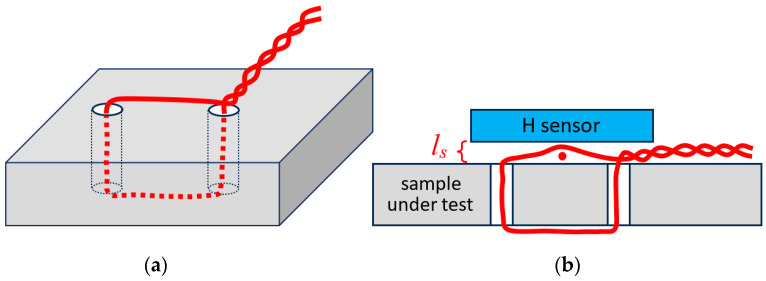
Example of a B-coil and its effect on an H-coil position: (**a**) single-turn B-coil; (**b**) two orthogonal B-coils crossing each other, causing the additional separation distance *l_s_* (cross-section view). The “dot” in sub-figure (**b**) shows the other orthogonal wire in this cross-sectional view.

B-coils are used for measurement of *B* in the sample under test. This requires drilling holes in the sample under test, and threading one or more turns through them (Figure 6a). However, for 2D or rotational magnetization measurements, there will be two orthogonal B-coils, whose wires cross in the middle. Therefore, the *additional* separating distance *l_s_* will be at least double the wire diameter used for the B-coils, and even more if multiple turns are used as practiced in the literature [3,6,10,11,13,18]. The alternative employed by several researchers is to use the *B* needle sensors, but they also have their drawbacks due to reduced sensitivity and troublesome avoidance of unwanted air flux in the sensing circuit [3,6,10].

For B-coils, even if relatively thin wires (0.1 mm diameter) are used, the minimum additional separation distance will be 0.2 mm, which is certainly not negligible for an H-coil whose nominal thickness is 0.75 mm. Worse still, if not accounted for, the B-coil wires constitute an unevenness of the surface onto which the H-coil is placed. This can lead to uneven elevation and further measurement errors caused by the *H* gradient as the local sensing is displaced at different distances from the sample surface.

**Figure 7:**
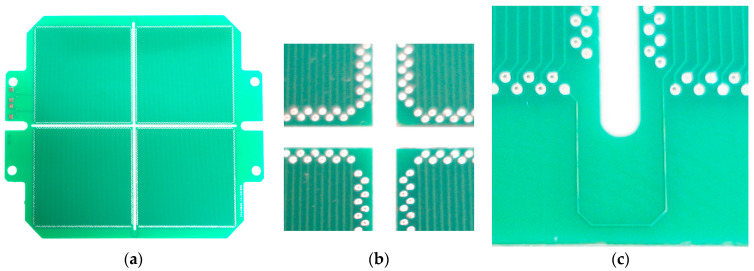
Prototype H-coil with machined-out channels for accommodation of B-coil wires: (**a**) overview; (**b**) close-up of the cross-shaped cutout in the middle; (**c**) close-up view of a bridging part.

This additional separation distance can be eliminated by using a special design as in the proposed H-coil prototype. Namely, the active sensing area was split into four square sub-regions, designed such that between them there are no conductors placed within the PCB. The PCB substrate was then machined out in a cross-shaped manner (Figure 7a) so that there is ample space provided for the B-coil wires, including their crossing in the middle (Figure 7b). The cut-out channel has a width of 1.5 mm, and it extends beyond the width of each H-coil (Figure 7c) so that the B-coil can have the same effective width as the H-coil, in order to synchronize their effective sensing areas. The surface of the H-coil can then be placed *directly* on the sample surface, even if the B-coil wires are relatively thick, or if there are multiple wire turns in them.

The four sub-regions are held together by bridging parts at the end of each arm of the cross-shaped cutouts (Figure 7c). All four sub-coils in the sub-regions are connected in series, so that they are combined into one sensing H-coil (active either in the X or in the Y direction, respectively). The connection to the next sub-region is fed around the bridging area (Figure 7c), and so is the return conductor, positioned exactly underneath, as per the concept illustrated in Figure 5b. Therefore, the bridging parts perform two tasks: mechanically connecting all sub-regions and providing an electrical path for the conductors that join the sub-regions. The same approach is used for both sub-coils (X and Y). The authors believe that this approach is presented in the literature for the first time.

## 5. Stackable Design

As mentioned above, in a general case there is a gradient of *H* amplitude above the sample surface. Therefore, sensing the value of *H exactly* at the sample surface is not possible due to the finite thickness of any *H* sensor. However, it was demonstrated experimentally that it is possible to extrapolate *H* values towards the sample surface if at least two sensors are used on top of each other [2,3,4,5]. The prototype comprises several mechanical features, particularly the four circular holes (Figure 3a and Figure 7a), which can be used for precise mechanical positioning of the sensor. Two sensors can be put directly on top of each other, and thus the same holes may be used for their positioning. Thickness-wise dimensions are definable with reasonable precision (Figure 4); therefore, such a stacked configuration can enable the extrapolation technique.

For such extrapolation of *H*, it is even more imperative that the additional spacing distance caused by the B-coil wires is not present, as it would harm the definition of precise distances that must be used in the calculation of extrapolation. The design presented in this paper allows complete elimination of the B-coil wire problem. The nominal thickness of 0.75 mm should provide ample space for the B-coil wires, and so the second H-coil positioned on the top of the first one need not require the cross-shaped cutout.

Of course, for an even better outcome extrapolation-wise, it would be advantageous to make fully integrated H-coils within a single PCB comprising appropriately more copper layers (e.g., at least eight layers). However, with such a design, it is no longer possible to use through-hole vias in the PCB so that the sub-coils would overlay on top of each other. But with more expensive technology (with so-called buried and blind vias), this is still possible. Also, with higher-capability technology, a higher density of turns can be achieved. This was not attempted in this project.

The design with each PCB containing just two sub-coils provides increased flexibility. For example, the effects of differences in the sensing plane elevations can be studied. But also the influence of the self-capacitance of the various sensor configurations can be investigated—this would not be possible with one fully integrated multiple-subcoil design.

## 6. Large H-Coil for Spatial Averaging of Magnetic Properties

The maximum size of the given H-coil is mostly dictated by the size of the 2D magnetizing yoke and/or the sample for which it is intended. In this case, the H-coil is intended for a circular sample with a diameter of 192 mm. This is why the square PCB has its side width (for sensing) of up to 116 mm, with the diagonal of 165 mm of the PCB shape, so that it fits in the available area and conforms to the uniformly magnetized area in the middle of the sample, well within the outer diameter [3,6,11,18].

The standardized SST method (single-sheet tester) [19] requires a minimum sample size of 30 cm × 45 cm = 1350 cm^2^ (taking into account just the distance between the inner sides of the yoke where the magnetization is uniform). Similarly, for the more industrially relevant Epstein frame standard [20], the minimum sample size depends on its weight, but for a typical electrical steel it can be as little as 12 strips, with the minimum uniform magnetizable area thus equal to 12 × 22 cm × 3 cm = 792 cm^2^ (excluding the corners operating at reduced magnetization). Lamination thickness can be assumed to be the same in each case; therefore, the surface area is used here as a proxy for the active volume of the sample.

Many international studies devoted to 2D and rotational magnetization utilized the area of uniform field only at the order of 2 cm × 2 cm = 4 cm^2^ [6,11,18], which is around 2.5 orders of magnitude less (200–340 times) than the minimum required for the Epstein frame or SST. Therefore, spatial averaging of the measured material properties could not take place to the same extent, and thus reproducibility of the method was also compromised, on top of other difficulties such as sensor orthogonality or feedback efficacy for waveshape control. A single large grain in grain-oriented electrical steel can have an area comparable to such a small sensing area [3,6], hence adequate averaging cannot take place (even if an ideally uniform H field is assumed). The size of the H-coil has no effect on the uniformity of the magnetic field, but the relative size ratio (H-coil vs. max. grain size) certainly will contribute to better averaging.

Even with the sensing area increased to 6 cm × 6 cm = 36 cm^2^ [21], the disparity is still at the level of 1.5 orders of magnitude (22–38 times) from the standardized methods. However, with the total sensing area increased to 11.3 cm × 11.6 cm = 131 cm^2,^ the ratio is narrowed down to just around one order of magnitude (6–10 times), and thus the achievable reproducibility has a much better chance of approaching those of the Epstein frame and the SST methods.

It should also be noted that the whole sample itself would be larger, at the level of 290 cm^2^. So, bearing in mind that the magnetic properties of the sample do not change abruptly outside of the sensor, a certain “overlap” of the properties can be expected to affect the detection area of the H-coil, because *H* is expected to be relatively uniform over the sensing area [2,3,10,21]. Therefore, in reality, the effective area responsible for “averaging” may be closer to 200 cm^2^ (160 mm diameter), thus giving a ratio of 4.5–6.8 times as compared to the standardized methods. This perhaps opens the path to obtaining sufficiently good reproducibility so that an international standardization of power loss measurement under rotational magnetization could be considered in the future [3], especially if sufficient reproducibility of the method can be demonstrated in interlaboratory comparison [6,11,18].

## 7. Estimation of Parasitic Self-Capacitance

As an inductive sensor, an H-coil represents some amount of inductance. The inductive and resistive values are directly measurable from the H-coil terminals with a suitable LCR meter, such as the Keysight UL1733 [22], which was used in this study. The resistance was measured with a DC function, and the inductance was measured at the highest available test frequency of *f* = 100 kHz. It was assumed that this measurement was suitable for the estimation of the order of magnitude of the parasitic effects, especially since measurements at higher frequencies could become affected by self-resonance so that the inductance reading could be falsified.

However, there is also a certain amount of parasitic (unwanted) self-capacitance contributed by turn-to-turn (and other effects) which is not directly measurable. Its order of magnitude is difficult to estimate, and therefore it should not be treated as “negligible”, as sometimes implied in the literature [18] (without providing an actual proof). For 2D and especially for rotational measurements, the *H* waveforms can be severely distorted (i.e., non-sinusoidal) [2,3,4,6,7,8,10,17,21,23], so much higher harmonics will be present in the waveforms. From a practical viewpoint, at least 30 harmonics can be expected at lower frequencies [2,3], perhaps somewhat fewer at higher frequencies [23], but the question of capacitance-affected bandwidth should not be treated as “negligible” under all conditions. Power frequency is 50–60 Hz, but as is commonly used in research, the tests can be up to 1–2 kHz [2,3,23]; hence, a rather high bandwidth, perhaps significantly exceeding 10 kHz, should be expected for accurate measurement of *H*. With PCB-based H-coils, the width of the track is wider (i.e., larger area) than the diameter of a typical wire that could be used for wire-wound H-coils. Also, there is an additional contribution from the even larger via pads, which may or may not significantly increase the parasitic capacitance. On top of all these factors, the large size and H-coil stacking (for extrapolation) can further contribute to self-capacitance. Therefore, the question of estimation of at least the order of magnitude of the self-capacitance was deemed important enough to be attempted experimentally, as described hereinbelow.

**Figure 8:**
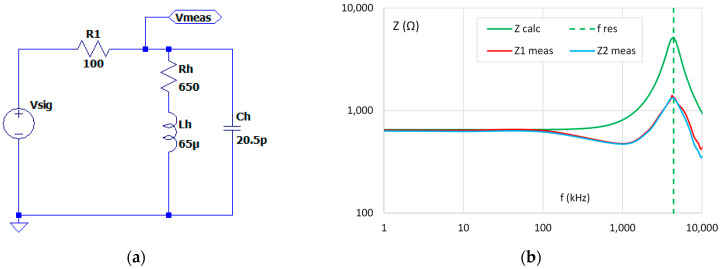
Estimation of parasitic capacitance: (**a**) simplistic bulk model; (**b**) experimental and analytically calculated results. In the range up to 100 kHz all the curves follow the same amplitude indicating the unaffected frequency bandwidth as discussed in the next section.

Due to the lack of any better information, a naïve, simplistic equivalent model of the H-coil parameters expressed as bulk quantities can be constructed as in Figure 8a. The measured DC resistance was *R_h_* = 650 Ω, inductance *L_h_* = 65 μH, and the self-capacitance was the parameter to be estimated from an otherwise known technique of self-resonance [2,3].

### 7.1. H-Coil Without External Conductors

The first test was carried out with the circuit, as shown in Figure 8a, where a sinusoidal signal generator was used as the supply (from 1 kHz to 10 MHz). The series resistor *R*_1_ (non-inductive) was used for current sensing, and the signal across the H-coil (*V_meas_*) and the voltage across *R*_1_ were measured with an oscilloscope. Both sensing coils (X and Y) were measured with the same setup, and the experimentally measured impedances *Z*_1_ *_meas_* and *Z*_2_ *_meas_* (respectively) followed the same curve shape, as should be expected (Figure 8b).

The data shown in Figure 8b were measured for the H-coil distanced from the surface (i.e., placed on a 10 mm thick block of an insulator), so that no external conductors were influencing the tests (from an additional capacitance viewpoint). From the measured inductance (assumed to be constant) and the frequency of the resonant peak (*f_res_* = 5.18 MHz), the self-capacitance was estimated as 20.5 pF.

Given all three parameters (L, R, C), the bulk model of Figure 8a was also used to analytically calculate the curve, and the equivalent calculated complex impedance *Z*_*calc*,*cplx*_ is as expressed by Equation (5a,b). The plot *Z_calc_* in Figure 8b is generated by using this equation.

(5a) $$Z_{calc,cplx}=\frac{R\cdot X_{C}^{2}}{R^{2}+{\left(X_{L}-X_{C}\right)}^{2}}-j\cdot \frac{X_{C}\cdot {{(R}^{2}+X}_{L}^{2}-X_{L}\cdot X_{C})}{R^{2}+{\left(X_{L}-X_{C}\right)}^{2}}$$

(5b) $$Z_{calc}=\left|Z_{calc,cplx}\right|$$

However, the resonant peaks coincide in Figure 8b, but there are additional inflection points in the experimental curves, which are not present in the data calculated for the over-simplistic model. Also, the amplitude at the resonance differs. This indicates that the simplistic model does not include all the parasitic components correctly.

The coil-to-coil directly measured capacitance was 2.94 nF, which is significantly larger than the estimated self-capacitance of each coil. Therefore, when the first coil was energized, it could be expected that there was some amount of capacitively coupled voltage difference imposed across the second coil and an additional impedance was thus added to the circuit. Because of the orthogonality of the coils, it is difficult to specify the character of such capacitive coupling (the coils are orthogonal, so no significant inductive coupling was expected).

**Figure 9:**
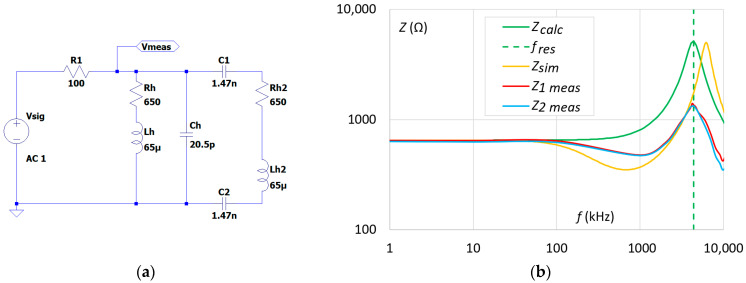
More complex bulk model for the dual H-coil: (**a**) bulk model; (**b**) experimental, analytical, and simulated results.

However, these values can be used naively for an adjusted bulk model, in which the total coil-to-coil capacitance is split into two equal values (*C*_1_, *C*_2_), and the *L_h_*_2_ and *R_h_*_2_ values for the second coil are the same as for the first (Figure 9a). The results for such a circuit were obtained with SPICE software (LTspice XVII), and the orange curve *Z_sim_* is shown in Figure 9b. The peak amplitude of impedance remains overestimated, but qualitatively the shape resembles the measured curve to a better degree, especially showing the dip around 1 MHz before the resonance. Qualitative resemblance cannot be treated as direct evidence, but this indicates that the behaviour of the dual coil is indeed more complex and non-negligible capacitive coupling between the coils does take place. But without additional conductors in the vicinity of such an H-coil, the effects do not appear to be practically significant up to 100 kHz (the impedance curves remain flat), which is a rather large bandwidth as compared to ordinary measurements on electrical steels, or similar soft magnetic materials. However, the plots in Figure 9b show only the amplitude variation, whereas the phase can also be affected, as shown in the next section.

### 7.2. H-Coil on a Conductive Plate

The default case for an H-coil is to be placed on a conductive plate such as electrical steel lamination. The presence of such a large conductive body near the coil has the potential to significantly increase the parasitic capacitance.

**Figure 10:**
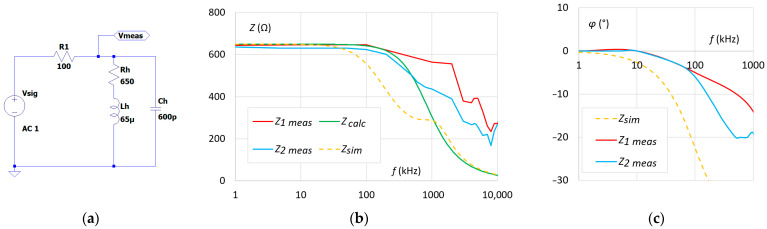
Results for the dual H-coil placed on a conductive plate: (**a**) simplistic bulk model. (**b**) Experimental, analytical, and simulated results for impedance amplitude. (**c**) Experimental and simulated results for phase shift. (These measurements were also carried out on a more conductive 10 mm thick aluminum plate, with very similar curves as (**b**). These results are not repeated here for clarity of the figure and brevity of the paper).

The test was repeated by placing the H-coil directly on a sheet of grain-oriented electrical steel, and the measured results were as in Figure 10b. This time, the two sensing coils (X and Y, denoted by *Z*_1_ *_meas_* and *Z*_2_ *_meas_*, respectively) differed by a larger amount, which could be expected due to their slightly different distance to the conductive sheet (refer also to Figure 4).

Interestingly, for this configuration the main resonant peak did not occur at all. A resonance also does not occur for the simpler analytical bulk model of Equation (5a,b) with an appropriately increased value of the parasitic capacitance. By trial and error, the capacitance *C_h_* was increased from 20.5 pF (Figure 8a) to 600 pF (Figure 10a), so that the droop of impedance amplitude coincided roughly with the decrease in the experimental curves above 100 kHz. With the larger capacitance, a resonant peak would be expected at a proportionally lower frequency, but the relative impact of the 650 Ω resistance was such that the system was overdamped and thus oscillations leading to a peak of impedance amplitude could not occur.

Note that for the resonance to take place, the imaginary component of impedance would need to equal zero. By rearranging the values from Equation (5a), the imaginary part can be presented as in Equation (6). For the numerator to equal zero, the component in the parentheses would have to equal zero. Even if *X_L_* were to be ignored, if *R* is greater than √(*L*/*C*), then the numerator cannot equal zero, and thus resonance cannot take place. As mentioned above, *R* = 650 Ω, *L* = 65 μH, and so with the estimated *C* = 600 pF, it would be √(*L*/*C*) = 329 Ω. Addition of always-positive *X_L_*^2^ will make the disparity even greater. Therefore, the cancellation to zero is not possible for such a circuit.

(6) $${Im\;(Z}_{calc,cplx})=-\frac{X_{C}\cdot {{(R}^{2}+X}_{L}^{2}-L/C)}{R^{2}+{\left(X_{L}-X_{C}\right)}^{2}}$$

However, again, in this case, the measured bandwidth is not practically affected up to 100 kHz, despite the fact that the estimated parasitic capacitance increased by a factor of 30 (from 20.5 pF to 560–600 pF).

But because of the simplistic model, Equation (5a,b) does not represent the shape of the measured curves qualitatively over the whole range. If the bulk model is adjusted as in Figure 9a, but with *C_h_* set to 560 pF, the SPICE model generates the dashed orange curve, *Z_sim_*, in Figure 10b. Because of the larger capacitances involved, the droop of the curve begins at a lower frequency (around 30 kHz), but there is a visible “shoulder” around 1 MHz, which is more akin to the shape of the experimental curves. For 560 pF, it would be √(*L*/*C*) = 341 Ω, which is still smaller than *R* = 650 Ω. The value of 560 pF was the largest at which the “shoulder” remained flat, rather than peaking with a local resonance.

It should be emphasized that the interaction between the two sensing coils (X and Y) is expected to be complex at least from the viewpoint of their mutual orthogonality. The potential difference in the first coil will be coupled across the width of the turns of the second coil, rather than along the sensing axis, so a complex interplay of spatially distributed parasitics is expected. This cannot be represented in the over-simplistic or even the less over-simplistic bulk model, as discussed above.

Notably, the phase curves (Figure 10c) show a departure from the ideal 0° level at and even below 100 kHz. The change is perhaps not large (around 5° at 100 kHz), but the departure seems to begin above 10 kHz, and thus can no longer be treated as completely “negligible” from a metrological viewpoint. It should also be emphasized that for the case of using two or more H-coils stacked on top of each other, *H* extrapolation will increase the overall parasitic capacitance even further. So it can be expected that even larger differences, at least in phase shift, can occur. Therefore, for a given measurement system, a specific arrangement of multiple H-coils should be investigated (for instance, at least with the method presented in this paper), especially if measurements at higher frequencies (>1 kHz) are to be carried out.

However, the goal of this exercise was not to measure the parasitic component precisely as such, but rather to illustrate qualitatively the practically relevant bandwidth of such a sensor, as carried out hereinabove. From experiments, it is clear that serious parasitic effects occur only above 100 kHz for such a coil; hence, under the conditions tested, the practical bandwidth could extend to such a range. The parasitic effects can be expected to become more pronounced for a system of stacked H-coils (stacked separate PCBs or combined into one PCB) due to proportionally increased self-capacitance and even more complicated inter-coupling (both capacitively and inductively).

## 8. Verification of Sensitivity Coefficient in a Long Solenoid

**Figure 11:**
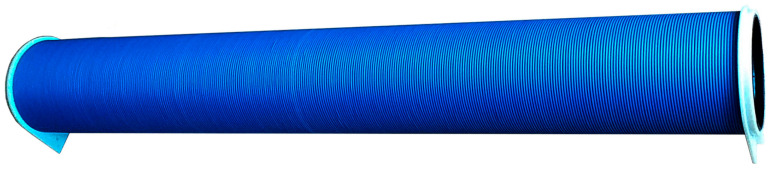
Large solenoid used for verification of sensitivity.

A large solenoid (Figure 11) was constructed for the purpose of experimental verification of the sensitivity of these large H-coils. The solenoid was wound on a tube with a 1.3 m length and 152.4 mm outer diameter. 3D printing was used to make flanges installed at each end of the tube, with the effective length between the inner surfaces of the flanges measured as 1214 mm after installation. Insulated wire with nominal outer diameter OD = 3.6 mm (measured with insulation) was used for a single layer of winding placed uniformly along the whole available length between the flanges, resulting in a total of 335 turns. This was slightly less than the ideal expected (1214 mm/3.6 mm = 337.2 turns) and could be caused by the local variation in the insulation thickness and distortions due to imperfect winding, even though the turns were touching each other throughout the length of the solenoid. These combined distortions would account for an average change in effective OD of +0.024 mm for each turn. Such a tolerance was within measurement accuracy of the relatively soft insulation of the wire.

Relatively thick insulated wire was used on purpose. If a thinner enamelled copper wire was used instead, then many more turns would fit into a single layer. The multiplicity of turns would increase the inductance of such a solenoid, and it would be more difficult to drive it from the compliance voltage viewpoint. In these experiments, the calibrator Transmille 3050 [24] was used, and, with such calibrators, it is known that in the current source mode it is not possible to drive objects with high impedance, due to compliance voltage limitations. Therefore, using a wire with a thicker insulation allows inherent “spacing” of the subsequent turns, but retaining good definition of their local position by virtue of the insulation thickness. So the uniformity of winding is reliably retained over the whole length, but without elevating the inductance to unacceptable values.

The diameter of the inner stranded copper core was estimated as 2.2 mm effective (not possible to measure a precise circle-equivalent value due to multiple strands). For calculation purposes, it could be assumed that the solenoid is either of a non-negligible thickness (“thick”, Equation (7)) or assumed to be “infinitely thin” (Equation (8)) [4]. Assuming simplistically that the solenoid was “infinitely long and thin”, Equation (8) simplifies to Equation (9).

(7) $$H_{thick}=\frac{I\cdot N_{s}}{\left(D-d\right)}\cdot \ln\frac{D+\sqrt{D^{2}+l^{2}}}{d+\sqrt{d^{2}+l^{2}}}$$

(8) $$H_{thin}=\frac{I\cdot N_{s}}{\sqrt{D_{0}^{2}+l^{2}}}$$

(9) $$H_{long}=\frac{I\cdot N_{s}}{l}$$

where *I*—applied current (A), *N_s_*—number of turns of the solenoid (unitless), *D*—outer diameter of the winding (m), *d*—inner diameter of the winding (m), *l*—length of the winding (m), *D*_0_—“average” diameter of the “infinitely thin” winding (m).

Assuming the “thick” solenoid as the reference value, the calculated magnetic field at the centre differs by less than 0.00006% for the “thin”, and up to 0.9% for the simple “long” equation (Table 1). For this reason, Equation (7) was used for verification of H-coils.

The verification of sensitivity was performed by measuring the output of the H-coil directly (no additional signal amplifiers) with a data acquisition module USB-6365 [25]. The voltage was integrated digitally in LabVIEW software, and the *H* value was calculated from Equation (4) assuming *NA* = 1 m^2^ (i.e., unity for *A* and for *N*). The solenoid current was supplied by a calibrator Transmille 3050 whose basic accuracy for AC current was 0.09% [24]. Then Equation (7) was used to calculate the solenoid’s *H*, and the sensitivity factor could be extracted by dividing the two values of *H*: from the solenoid and from the H-coil (with assumed *NA* = 1 m^2^).

The effective width in Table 2 for each sub-coil is a sum from two rectangles, because the inner cross-shaped cutout needs to be excluded. The dimensions were taken as the average between the two rows of vias (e.g., see Figure 7c).

The *NA* values estimated from the PCB prototype are very close to the real performance (better than 2%), but it is clear that, for precise measurement, the calibration would certainly be required. Interestingly, the agreement between the expected and the actual values is very good, whereas for the wound coils the discrepancy can reach even tens of percent (especially for thin coils) [3].

## 9. Summary and Conclusions

This project demonstrated that it is possible to manufacture large H-coils (with a sensing area of 116 mm × 113 mm × 0.75 mm and a PCB size of 165 mm diagonally) so that their construction is designed to comprise multiple metrologically relevant features simultaneously. Most of these multiple features were previously described in the literature separately, but for the first time, they were combined in a single physical sensor.

With the PCB design, the orthogonality of sensing can be controlled for each and every turn of the coil, so it is expected that a higher degree of orthogonality can be achieved than is possible with wire-wound H-coils. However, experimental verification of orthogonality was not possible at this stage. PCB-based thin and stable sensors can be produced with relative ease and at relatively low cost, with a nominal thickness of 0.75 mm demonstrated here; even thinner designs can be easily and reliably obtained with ordinary PCB technology (0.5 mm was demonstrated in the past).

PCB design allows splitting the H-coil surface into multiple sensing sub-sections positioned in a special controlled way within the available area. This can provide room for cutouts within the board so that the space for B-coil wires can be accommodated. This enables elimination of the extra distance to the sample surface, and the H-coil can be positioned directly on the sample surface, improving reproducibility of sensor placement and accuracy of defining dimensions. Moreover, the mechanical design was executed to provide mechanical features for a controlled, stackable design and for the extrapolation of *H* towards the sample surface.

It is believed that this study demonstrated for the first time (for H-coils) an experimental estimation of the order of magnitude of the parasitic self-capacitance of the sensing coils. With the prototype H-coil by itself, the estimated value was 20.5 pF for one sensing coil. But with the H-coil placed on a conductive plate, the estimated value of self-capacitance was on the order of 600 pF, and the bandwidth appeared to be affected by the even larger coil-to-coil capacitance, which was measured directly at 2.94 nF. The measurements and estimations of the parasitics indicate that indeed the ordinary sensing bandwidth in the area of interest is unlikely to be affected by the self-capacitance, because such H-coils are intended for measurements at power frequencies (50 or 60 Hz nominal), and measurements at the fundamental frequency beyond 10 kHz are typically not possible due to magnetizing yoke limitations. It was shown that, for the H-coil performance itself, the bandwidth should not be affected up to 100 kHz (even in the immediate vicinity of the metal plate of the sample under test). The bandwidth should be expected to be limited to a lower frequency if stacked H-coils are used for extrapolation of *H* towards the sample surface.

The sensitivity factor is dictated by the *AN* product. For 229 turns, this was estimated as 0.011693 m^2^ and 0.012087 m^2^ for the X and Y sensing coils, respectively. Calibration in a purposely built long solenoid (1.3 m length, 163 mm diameter, 335 turns) driven with a calibrator in an AC current mode returned sensitivity values as 0.011903 m^2^ and 0.012169 m^2^, which agreed with those estimated to −1.76% and −0.67%, respectively.

## Figures and Tables

**Table 1:** Calculated values of the magnetic field strength at the centre of the solenoid.

Parameter	Thick Solenoid	Thin Solenoid	Long Solenoid
*N_s_*	335	335	335
*D*	165.2 mm	-	-
*D*_0_	-	163.0 mm	163.0 mm
*d*	160.8 mm	-	-
*l*	1214 mm	1214 mm	1214 mm
Equation	(7)	(8)	(9)
Calc. *H* at 1 A	273.4929 A/m	273.4931 A/m	275.9473
Difference	(Reference)	+0.00006%	+0.9%

**Table 2:** Calculated, estimated, and calibrated parameters for the H-coil prototype.

Parameter	H-Coil X	H-Coil Y
*N*	229	229
Coil width *	2 × 55.5 mm = 111.0 mm	2 × 57.0 mm = 114 mm
Thickness *	0.460 mm	0.463 mm
*A* *	51.060 mm^2^	52.782 mm^2^
*NA* *	0.011693 m^2^	0.012087 m^2^
*NA* (from calibration)	0.011903 m^2^	0.012169 m^2^
Diff. estimated vs. calibrated	−1.76%	−0.67%

* Estimated from PCB dimensions.

## Data Availability

Additional data may be available upon reasonable request.
